# Feasibility of intraprocedural integration of cardiac CT to guide left ventricular lead implantation for CRT upgrades

**DOI:** 10.1111/jce.14896

**Published:** 2021-02-10

**Authors:** Justin Gould, Baldeep S. Sidhu, Benjamin J. Sieniewicz, Bradley Porter, Angela W. C. Lee, Orod Razeghi, Jonathan M. Behar, Vishal Mehta, Mark K. Elliott, Daniel Toth, Ulrike Haberland, Reza Razavi, Ronak Rajani, Steven Niederer, Christopher A. Rinaldi

**Affiliations:** ^1^ Department of Cardiology Guy's and St Thomas' NHS Foundation Trust London UK; ^2^ School of Biomedical Engineering and Imaging Sciences King's College London UK; ^3^ Medical Imaging Technologies Siemens Healthineers Malvern Pennsylvania USA

**Keywords:** cardiac CT, CRT, image guidance, improving CRT response

## Abstract

**Background:**

Optimal positioning of the left ventricular (LV) lead is an important determinant of cardiac resynchronization therapy (CRT) response.

**Objective:**

Evaluate the feasibility of intraprocedural integration of cardiac computed tomography (CT) to guide LV lead implantation for CRT upgrades.

**Methods:**

Patients undergoing LV lead upgrade underwent ECG‐gated cardiac CT dyssynchrony and LV scar assessment. Target American Heart Association segment selection was determined using latest non‐scarred mechanically activating segments overlaid onto real‐time fluoroscopy with image co‐registration to guide optimal LV lead implantation. Hemodynamic validation was performed using a pressure wire in the LV cavity (dP/dt_max)_).

**Results:**

18 patients (male 94%, 55.6% ischemic cardiomyopathy) with RV pacing burden 60.0 ± 43.7% and mean QRS duration 154 ± 30 ms underwent cardiac CT. 10/10 ischemic patients had CT evidence of scar and these segments were excluded as targets. Seventeen out of 18 (94%) patients underwent successful LV lead implantation with delivery to the CT target segment in 15 out of 18 (83%) of patients. Acute hemodynamic response (dP/dt_max_ ≥ 10%) was superior with LV stimulation in CT target versus nontarget segments (83.3% vs. 25.0%; *p* = .012). Reverse remodeling at 6 months (LV end‐systolic volume improvement ≥15%) occurred in 60% of subjects (4/8 [50.0%] ischemic cardiomyopathy vs. 5/7 [71.4%] nonischemic cardiomyopathy, *p* = .608).

**Conclusion:**

Intraprocedural integration of cardiac CT to guide optimal LV lead placement is feasible with superior hemodynamics when pacing in CT target segments and favorable volumetric response rates, despite a high proportion of patients with ischemic cardiomyopathy. Multicentre, randomized controlled studies are needed to evaluate whether intraprocedural integration of cardiac CT is superior to standard care.

## 1 INTRODUCTION

Patients with heart failure and pre‐existing pacing or implantable cardioverter‐defibrillator (ICD) systems may benefit from cardiac resynchronization therapy (CRT) upgrade,^1^ however suboptimal left ventricular (LV) lead placement outside of late activating regions and in myocardial scar may result in suboptimal response.^2^ Cardiac magnetic resonance (CMR) can guide LV lead placement targeting late mechanical activation (LMA) and avoiding LV scar.^3^ However, 28% of CRT candidates have pre‐existing pacing or ICD systems and may be unsuitable for CMR,^4^ which is not without risk if patients are pacing dependent. Furthermore, patients with heart failure often find CMR challenging due to long breath holds or prolonged supine periods (30–45 min) and image degradation from lead artifact impedes its utility.

Cardiac computed tomography (CT) has the potential to guide LV lead placement.^5^, ^6^ Rapid acquisition of isotropic 3‐dimensional (3D) whole heart data sets with submillimetre spatial resolution allows accurate assessment of coronary venous anatomy,^5^ regional or global LV systolic function assessment^5^, ^7^ and evaluation of LV dyssynchrony or LMA.^5^, ^8^ Additionally, CT may detect regional hypoperfusion and myocardial scar^9^ albeit with varying results with no current standardized imaging protocols to reliably identify late iodine enhancement.^10^

We have previously shown that “offline” preprocedural cardiac CT dyssynchrony analysis produces functional data sets with sufficient temporal resolution to differentiate LMA segments in a separate cohort of 18 patients and that CT target selection retrospectively correlated well with acute hemodynamic response (AHR).^5^ We previously showed the utility of “real‐time” X‐magneic resonance imaging (MRI) guidance for CRT,^3^ however, to date stand‐alone Preprocedural cardiac CT with intraprocedural image integration to guide LV lead placement has not been demonstrated.

We set out to test the feasibility of a purpose‐built, integrated software platform to process, analyze and overlay CT data within a cardiac catheter laboratory to prospectively guide LV lead implantation. To achieve this, we performed preprocedural cardiac CT with intraprocedural image integration to target LV lead placement with acute hemodynamic validation.

## 2 METHODS

Between September 2017 and August 2019, 18 patients with a prior pacemaker or ICD undergoing CRT upgrade were prospectively enrolled. All patients provided written informed consent. The study protocol was approved by the West Midlands Research Ethics Committee (REC:14/WM/1069) and conducted in accordance with the Declaration of Helsinki.

### 2.1 Recruitment and follow‐up

Consecutive patients ≥18 years of age with heart failure and LV ejection fraction (LVEF) less than 40% undergoing CRT upgrade were eligible if they met all study requirements, were on optimal heart failure pharmacotherapy for ≥3 months Before enrollment and could provide informed consent. Patients were ineligible if eGFR < 30 ml/min/1.73 m^2^, previous iodine contrast allergy or any contraindication to CRT/transvenous LV lead implantation via the coronary sinus (CS). All patients underwent the following tests at baseline and 6‐month follow‐up visits post CRT upgrade: New York Heart Association (NYHA) functional class assessment; physical examination; 12‐lead resting ECG; two‐dimensional transthoracic echocardiogram including Simpson's biplane LV end‐diastolic volume (LVEDV), LV end‐systolic volume (LVESV) and LVEF; Minnesota living with heart failure questionnaire score (MLWHFQ); 6‐min walk test (6MWT).

### 2.2 Preprocedural cardiac computed tomography dyssynchrony imaging and analysis

**Figure 1:**
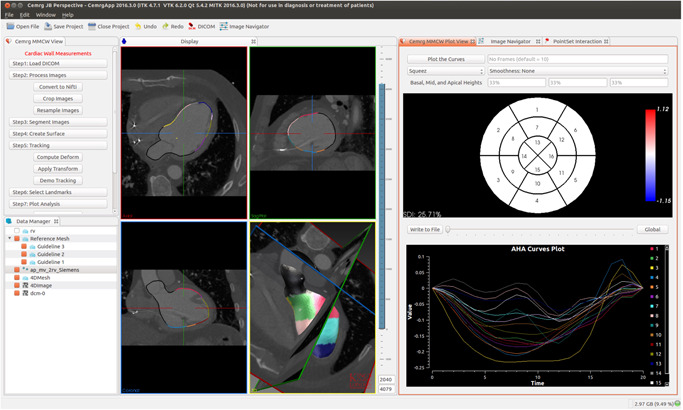
Cardiac computed tomography (CT) yssynchrony analys is platform based on open‐source software medical imaging interaction toolkit (MITK) provides a simple stepwise approach for tracking wall motion from cardiac CT datasets. (A) Interactive image rendering for visualizing 3D images and surface meshes. (B) 16‐segment bullseye plot for visualization of myocardial strain. (C) Individual strain curves; each color represents an American Heart Association (AHA) segment

All patients underwent dedicated cardiac CT during RV pacing Before CRT upgrade using a 3rd generation dual source scanner (SOMATOM Force; Siemens Healthineers) with temporal resolution up to 66ms. Intravenous metoprolol helped achieve a heart rate less than 65 beats/min in sinus rhythm and less than 100 beats/min in atrial fibrillation. A topogram was used for localization and automatic exposure control. Following injection of 120 ml iodinated contrast material (Omnipaque 350 mg/ml iodine; GE Healthcare) at 5 ml/s via the antecubital vein, a retrospective ECG‐gated cardiac CT angiography (CTA) was performed with reference dose settings 100 kV and 288 mAs/rot. Contrast monitoring triggered the scan with 14 s delay after reaching 100HU (at 100 kV) in the descending aorta. Full cardiac function was acquired for motion analysis throughout the cardiac cycle (0%–100%, 5% increments) and coronary venous anatomy for identifying target veins subtending target segments. Comprehensive cardiac CT dyssynchrony assessment was adapted from Behar et al.^5^ and calculated using opensource software (Figure 1). Image registration warping field was applied to a triangulated LV endocardial mesh from ECG‐gated cardiac CT angiograms. Endocardial wall motion was tracked using single semi‐automated LV cavity segmentation at end‐diastole with motion characterized by circumferential and longitudinal strains as well as local area change throughout the cardiac cycle. Derived motion was validated against manually annotated anatomical landmarks and strain calculations were verified using idealized problems.

**Figure 2:**
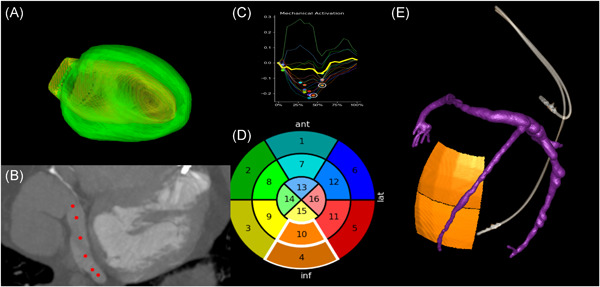
Pre‐implant CT Guided CRT workflow. (A) Automatic segmentation of LV epicardium and endocardium to create 3D LV mesh. (B) Semi‐automatic segmentation of coronary venous anatomy using intermittent 3D markers (red circles) generates 3D reconstruction of coronary sinus (CS)/veins. (C) Integration of CT‐derived dyssynchrony plots allows selection of latest mechanically activating segment. (D) Target selection on AHA 16‐segment bullseye plot using dyssynchrony curves. Latest mechanically activating segments (time to peak contraction) without LV scar defined the optimal target segments for LV lead delivery. Septal and minimal endocardial strain segments were excluded as likely represent regions of nonviability. 11 Each color represents an AHA segment. (E) 3D fusion of target AHA segments with CS segmentation to identify target veins subtending the target segment. A large posterolateral vein subtends basal‐mid inferior segments in this example. AHA, American Heart Association; CRT, cardiac resynchronization therapy; CT, computed tomography; LV, left ventricular

Before the LV lead implant procedure, target American Heart Association (AHA) segment selection was performed using the latest mechanically activating segments (identified using time to peak contraction, Figures 1C and 2C) outside of visible or inferred scar (inferred by wall thinning, hypoattenuation or akinetic myocardium on retrospective cardiac CT). Septal and minimal endocardial strain segments were considered to represent regions of nonviability and were excluded from target selection.

### 2.3 Pre‐implant cardiac CT scar imaging

The initial eight patients underwent end‐systolic prospectively ECG‐triggered late enhancement scanning with a dual energy scan 7 min after contrast injection. For patients in sinus rhythm with hazards ratio (HR) less than 65 beats/min, scanning was performed at 90/Sn150 kV with 165 and 127 mAs reference, respectively with a full 250 ms reconstruction. A single energy shuttle mode dynamic scan acquiring for 15 s (4–5 cycles) at 80 kV/300 mAs (reference dose settings) was used for patients with atrial fibrillation and/or HR > 65 beats/min. The last 10 patients at 12.5 min following contrast injection underwent single energy shuttle mode dynamic scanning regardless of heart rate or rhythm with reference doses increased by 30% to reference kV 80 and reference mAs 390. Late iodine enhancement images were reconstructed using 2 mm slice thickness and 1 mm increments with medium smooth kernel (Qr36) and iterative correction of iodine beam hardening. The dynamic scan time points were averaged after nonrigid registration. The resulting average volume was loaded into a DICOM viewer and qualitatively evaluated in apical, mid, and basal short‐axis slices in a narrow window. For comparison, systolic phases were loaded in synchronized orientation.

### 2.4 Intraprocedural image overlay and LV lead guidance using “guide CRT” platform

**Figure 3:**
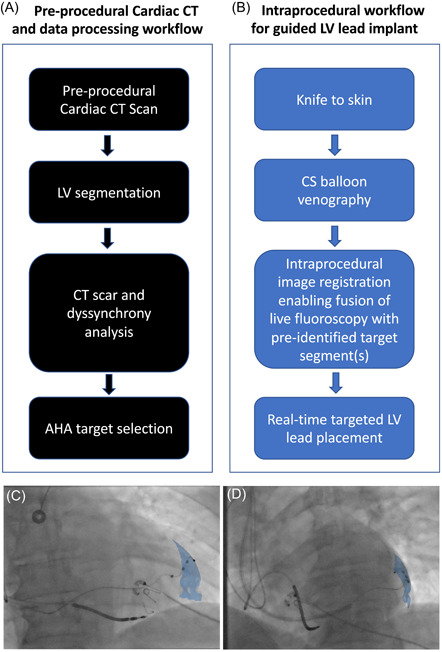
Guide CRT workflows. (A–C) Preprocedural data processing (A) and intraprocedural (B) workflows. (C–D) Final LV lead position with LV lead deployed in mid‐anterolateral AHA (blue) target segment represented in (C) posterior‐anterior and (D) left‐anterior‐oblique 30 degree projections. AHA, American Heart Association; CRT, cardiac resynchronization therapy; LV, left ventricular

Image overlay using Guide CRT (Siemens Healthineers) and CMR‐derived target segments has previously been described.^3^, ^12^ Guide CRT is a custom‐built software prototype on a dedicated workstation, integrated into an Artis‐Q biplane fluoroscopic angiography system (Siemens Magnetom Artis Combi Suite; Siemens Healthcare GmbH). Rapid, automatic data processing allows information to be extracted from cardiac CT images with an automated protocol for slice registration and LV segmentation. Cardiac CT acquisition, scar/dyssynchrony evaluation and identification of the optimal target AHA segments were performed 2–4 weeks before LV lead implantation by an experienced cardiologist with expertize in cardiac CT and uploaded to the guide CRT platform (Figures 2 and 3A). CT‐derived AHA target segments were co‐registered and overlaid onto fluoroscopic images to guide optimal LV lead delivery (Figure 3B).

### 2.5 LV lead implant and hemodynamic assessment

Following CS cannulation, occlusive balloon venography was performed in three fluoroscopic projections (right‐anterior‐oblique 30°; postero‐anterior; left‐anterior‐oblique 30°). Fluoroscopic images were fused with the CT‐derived 3D mesh and target segments subtended by target coronary veins (Figure 3B). Bony landmarks, existing pacing wires ± sternotomy wires facilitated image fusion and motion compensation. LV lead placement to multiple veins (target and nontarget segments) was performed and hemodynamic assessment undertaken with invasive dP/dt_max_ measurements using a 0.014‐inch high‐fidelity “wireless” Pressure Wire X (St. Jude Medical) in the LV cavity via a retrograde arterial approach as previously described.^13^ LV‐dP/dt_max_ measurements were recorded at each pacing site as previously described,^13^ using CoroFlow (Coroventis). Results were expressed as percentage change between baseline and biventricular pacing (AHR). Pressure wire readings did not dictate final LV lead positions which were based on CT‐derived target segments (Figure 3C,D) where coronary venous anatomy allowed with target thresholds of less than 2.5 V at 0.5 ms and absence of phrenic nerve stimulation (PNS). A quadripolar LV lead was used in all cases ensuring the cathode was within the target segment where possible. In absence of target veins subtending the target segments, the next adjacent vein to the target segment was used.

### 2.6 Statistical analysis

Discrete data are presented as *n* values with corresponding percentages in parentheses and continuous data as mean ± 1 *SD* or median[interquartile range]. Discrete variables were compared using Fisher's exact test. Normally distributed data were compared with a paired samples *t* test. Non‐normally distributed data were compared using Wilcoxon signed‐rank testing. For all tests, *p* ≤ .05 was considered significant. Analyses were performed using Statistical Package for Social Sciences, Macintosh V24.0.0.1(2017).

## 3 RESULTS

Eighteen patients underwent CT dyssynchrony and scar assessment. Baseline characteristics are summarized in Table 1. Patients were predominantly male (94.4%), with an RV pacing burden of 60.0 ± 43.7%, RV paced QRS duration of 154 ± 30 ms and 10 out of 18 (55.6%) had ischemic cardiomyopathy.

**Table 1:** Baseline characteristics

**Characteristic**	**Value**
Age (years)	67.0 ± 9.9
Male gender	17 (94.4)
Ischemic cardiomyopathy	10 (55.6)
NYHA III/IV	7 (38.9)
MLWHF questionnaire score	36.6 ± 23.5
6MWT distance (m)	316 ± 128
NT‐proBNP (ng/ml)	1108.1 ± 767.3
Hemoglobin (g/L)	139.4 ± 13.8
eGFR (ml/min/1.73m^2^)	69.1 ± 21.0
Paced QRS duration (ms)	154 ± 30
Left bundle branch block	15 (83.3)
CT Dose Length Product (mGycm)	1196 (1069–2049)
Atrial fibrillation	9 (50)
RV pacing burden (%)	60.0 ± 43.7
RV pacing burden >40%	18 (100)
ACE inhibitor/ARB/Sacubitril and Valsartan	18 (100)
Beta‐blocker	16 (88.9)
Aldosterone antagonist	12 (66.7)
Loop diuretic	9 (50)
Antiarrhythmic	4 (22.2)
Antiplatelet	7 (38.9)

*Note*: Values are presented as mean ± *SD*, median (IQR) or as *n* (%).

Abbreviations: 6MWT, 6‐min walk test; ACE, angiotensin converting enzyme; ARB, angiotensin receptor blocker; CT, computed tomography; eGFR, estimated glomerular filtration rate; IQR, interquartile range; MLWHF, Minnesota living with heart failure; NT‐proBNP, N‐terminal pro‐B‐type natriuretic peptide; NYHA, New York Heart Association.

### 3.1 Cardiac CT planning outcomes and scar identification

**Figure 4:**
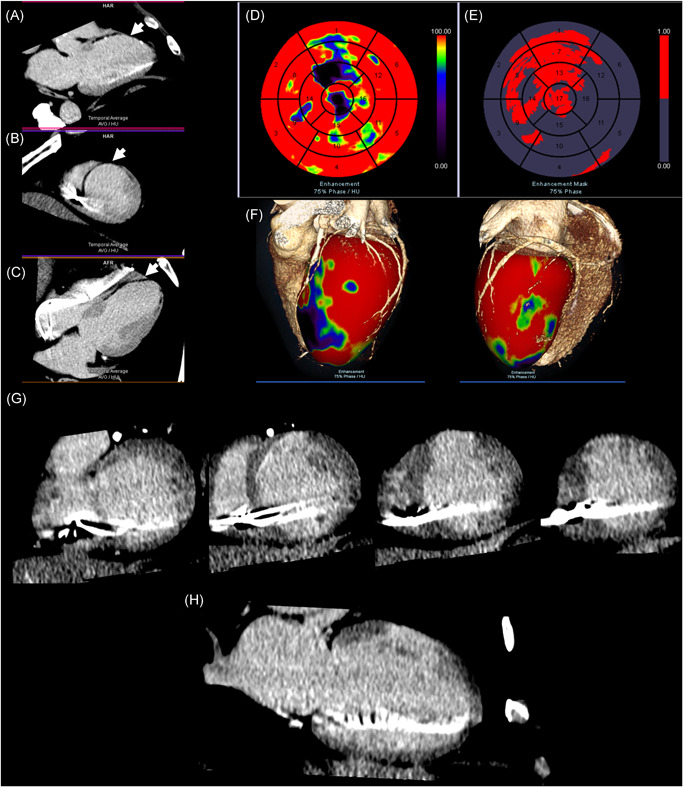
Cardiac CT scar analysis. Images acquired with dynamic single‐energy cardiac CT, 12.5 min post‐contrast administration. (A) Three‐chamber acquisition showing hypoattenuation (white arrow) in basal‐anteroseptal, mid‐anteroseptal and apical‐anterior segments. (B) Short‐axis slice showing hypoattenuation (white arrow) in mid‐anterior and anteroseptal segments. (C) Two‐chamber acquisition slice showing hypoattenuation (white arrow) in basal‐apical anterior segments. (D) AHA 17‐segment LV polar plot showing first‐pass enhancement mapping. Red represents high CT values ≥100HU with good first‐pass enhancement. Purple/blue represents CT values 0–75HU with less contrast in first‐pass enhancement. (E) AHA 17‐segment LV polar plot showing enhancement mask mapping. Red represents hypodense myocardial areas in first‐pass enhancement (relatively low contrasted regions). (F) Three dimensional volume‐rendered cardiac CT angiography images and first‐pass enhancement map fused with LV endocardial mesh. Color spectra as per (D). (G) Series of short‐axis slices showing late iodine enhancement in mid‐apical anterior segments, extending into mid‐anterolateral segment in keeping with prior left‐anterior‐descending artery territory infarction. (H) Single two‐chamber slice showing transmural late iodine enhancement in mid‐anterior segment. AHA, American Heart Association; CT, computed tomography; LV, left ventricular

All 18 CT scans were completed successfully with median radiation dose length product of 1196 (interquartile range [IQR], 1069–2049) mGycm and mean supine CT scan time of 14.3 ± 2.0 min. CT post‐processing (scar and dyssynchrony assessment) time was 23.9 ± 7.4 min (combined CT scan and processing time 38.2 ± 6.0 min). All (10/10) patients with ischemic cardiomyopathy had CT demonstration of scar; Late iodine enhancement was observed in two patients and regional hypoperfusion (seen as hypoattenuation) in keeping with myocardial scar in two further patients (Figure 4 and Table SA1). These correlated with regional late gadolinium enhancement (LGE) from historical CMR imaging. A further six patients had LV scar inferred by myocardial wall thinning and regional hypokinesis/akinesis on CT without identifiable late iodine enhancement or hypoperfusion. Post‐processing CT analysis identified proposed target AHA segments outside of scar subtended by target coronary veins in all 18 patients.

### 3.2 CT guided CRT implantation

Seventeen out of 18 (94%) patients underwent successful LV lead implantation (one patient had unsuccessful LV lead implantation due to an acute CS angulation preventing intubation and subsequently underwent leadless LV endocardial pacing). LV lead delivery to CT‐derived target vein(s) subtending target AHA segments was successful in 16 out of 18 (89%) patients (Table 2) and the LV lead (cathode) was deployed in the CT target segment in 15 out of 18 (83%) patients (Table 2). LV lead deployment to the CT target was not possible in two patients: one due to PNS with all available vectors and another the target vein was too small to pass a lead. Mean time from CS intubation to final LV lead deployment was 59 ± 26 min and complete implant procedural time (skin incision to closure) was 142 ± 47 min. Mean fluoroscopy time was 28.2 ± 13.4 min, median fluoroscopy radiation dose area product was 1444 (IQR, 947–2170) cGycm^2^ and mean implant contrast dose was 86.1 ± 49.4 ml. Total CT guided procedure time including CT scan, post‐processing and implant time was 172.7 ± 59.6 min. There was one procedural complication of pericardial effusion with hypotension successfully treated with pericardial drainage secondary to difficult ICD lead placement within the right ventricle with no long‐term sequelae.

**Table 2:** Feasibility of CT guided CRT

**Variable**	**Value**
Feasibility of CT guided LV lead placement in target vein	16/18 (89)
Feasibility of CT guided LV lead placement in target AHA segment^a^	15/18 (83)
All‐cause mortality	0
Heart failure hospitalization	0
Other cardiovascular hospitalization	2/18 (11)^b^
Intraprocedural related complications	1/18 (5.6)^c^

*Note*: Values are presented as *n* (%). Feasibility of using real‐time cardiac CT image overlay guidance, placing the LV lead in the CT‐derived target vein and target segment and maintaining CRT pacing at 6 months.

Abbreviations: AHA, American Heart Association; CRT, cardiac resynchronization therapy; CT, computed tomography; ICD, implantable cardioverter‐defibrillator; LV, left ventricular.

^a^ cathode placed in target segment

^b^ One patient was admitted with angina treated with optimization of anti‐anginal medication. Another patient with unsuccessful LV lead implantation was admitted electively for leadless LV endocardial pacing system (WiSE‐CRT, EBR systems).

^c^ One procedural complication of pericardial effusion with hypotension successfully treated with pericardial drainage secondary to difficult ICD lead placement within the right ventricle with no long term sequelae.

### 3.3 Validation of CT target segments with the acute hemodynamic response

Of the 17 patients with successful LV lead implantation, hemodynamic data was recorded in 14 patients. Three patients did not receive a pressure wire to obtain hemodynamic data: one due to poor patient compliance and two due to RADI analyzer malfunction. AHR was compared within CT target versus nontarget segment pacing sites (mean 2 ± 1 coronary veins per patient). In two patients, only a single target vein (with no comparative AHR in a nontarget segment) was achievable due to unfavorable coronary anatomy and therefore not included in comparative AHR analysis. An average AHR > 10% was considered a positive result and was achieved in 10 out of 12 (83.3%) CT target segments compared to 3 out of 12 (25.0%) nontarget segments (*p* = .012). Percentage change in dP/dt_max_ from baseline to biventricular pacing was significantly higher within CT target segments versus nontarget segments (14.4 ± 7.4% vs. 6.9 ± 5.9%, *p* < .001) (Figure SA2).

### 3.4 Follow‐up

Echocardiographic and clinical measures at baseline and 6‐month follow‐up are shown in Table 3. LVESV was improved at 6 months compared to baseline (133.8 ± 67.7 vs. 103.5 ± 53.9 ml; *p* = .003). Furthermore, LVEDV, NYHA functional class, and paced QRS duration were significantly lower at 6 months compared to baseline (Table 3). MLWHFQ scores, 6MWT distance, and NT‐proBNP were similar at 6 months follow‐up compared to baseline (Table 3). Echocardiography with image quality suitable for remodeling assessment was available in 15 patients. For these patients, volumetric response (LVESV improvement >15%) occurred in 9 out of 15 (60.0%) patients. Comparing HF etiology, in the ICM group remodeling occurred in 4 out of 8 (50.0%) versus 5 out of 7 (71.4%) in the NICM group (*p* = .608).

**Table 3:** CRT response—echocardiographic and clinical measures at baseline and 6‐month follow‐up

**Variable**	**Baseline**	**6‐Month follow‐up**	***p* Value**
LVEDV (ml)	200.8 ± 75.8	178.0 ± 63.2	0.028
LVESV (ml)	133.8 ± 67.7	103.5 ± 53.9	0.003
LVEF (%)	36.2 ± 9.4	44.2 ± 11.2	0.038
Paced QRS duration (ms)^a^	154 ± 30	134 ± 21	0.016
NYHA functional class	2.1 ± 0.6	1.5 ± 0.7	0.031
MLWHFQ score	30.9 ± 23.6	27.3 ± 24.2	0.266
6MWT distance (m)	341.8 ± 124.5	383.8 ± 138.1	0.178
NT‐proBNP (pg/ml)	1161.6 ± 903.9	1163.4 ± 1435.9	0.997

*Note*: All values are presented as mean ± *SD*. Absolute and percentage change values are the difference between values obtained from baseline pre‐assessment visit and 6‐month follow‐up.

Abbreviations: 6MWT, 6‐min walk test; CRT, cardiac resynchronization therapy; LVEF, left ventricular ejection fraction; LVESV, left ventricular end‐systolic volume; MLWHFQ, Minnesota living with heart failure questionnaire; NT‐proBNP, N‐terminal pro‐B‐type natriuretic peptide; NYHA, New York Heart Association.

^a^ QRS duration reflects the change in QRS duration from baseline to CRT at 6‐month follow‐up.

## 4 DISCUSSION

This study demonstrates for the first time the feasibility of intraprocedural image overlay of CT‐derived scar and dyssynchrony to guide LV lead implantation.

We have shown that:

(1) CT imaging was possible with demonstration of scar in all ischemic patients
(2) Image overlay of CT target segments was feasible allowing intraprocedural LV lead guidance
(3) Pacing within CT target segments showed superior AHR compared to nontarget segments confirming the validity of this approach.

The present study builds upon previous work validating “offline” retrospective CT dyssynchrony analysis to identify LMA segments as potential targets for LV lead placement that correlated well with invasive AHR data.^5^ In the present prospective study, we have demonstrated the feasibility of intraprocedural CT image overlay guidance to target LMA segments outside of scar with successful LV lead placement within CT‐derived target segments in 83% of patients. In addition, LV stimulation in CT targets were more likely to have an AHR > 10% than nontarget segments, a metric shown to predict chronic reverse remodeling.^13^ A notable finding was the ability to infer the presence of scar in all ischemic patients and the ability to avoid LV lead implantation to scarred regions may have significant clinical importance.

### 4.1 Feasibility of intraprocedural CT image overlay guidance

**Figure 5:**
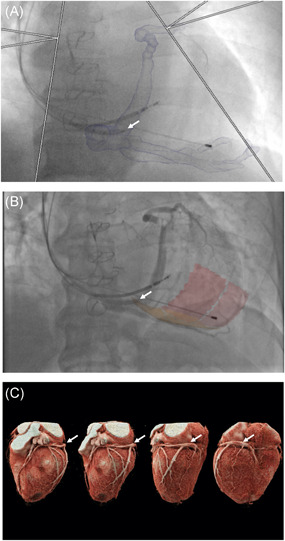
CT‐derived coronary venous anatomy. (A) Overlaid onto fluoroscopy to aid operator CS cannulation. Guide catheter (arrowed) entering CS ostium. (B) Balloon venogram of corresponding coronary venous anatomy with target segments overlaid onto fluoroscopy. (C) Volume‐rendered cardiac CT angiography series delineating coronary venous anatomy (arrowed). CS, coronary sinus; CT, computed tomography

Mean time from CS intubation to final LV lead deployment was 58 ± 26 min allowing for invasive acute hemodynamic data acquisition with pacing in multiple coronary veins, which significantly lengthened implant time. The combined CT scan or data processing time was 38.2 ± 6.0 min which is likely to improve with future software iterations and full automation. The main disadvantage of additional ionizing radiation may potentially be offset by improved preprocedural planning of target segments and intraprocedural image overlay guidance of coronary venous anatomy and CS ostium location (Figure 5). This study used an LV cavity pressure wire to validate CT guided lead targets. Accessing the LV cavity increases procedural complexity with risk of arterial injury and thromboembolism; however, the pressure wire should not be required in future CT guided studies.

### 4.2 Comparison with previous studies

Zhou et al.^14^ reconstructed 3D LV venous anatomy from dual‐view fluoroscopic venograms fusing it with LV epicardial surfaces on SPECT MPI. Sommer et al., using integrated CT‐derived CS anatomy, ^99m^Technetium myocardial perfusion imaging and speckle‐tracking echocardiography radial strain, targeted optimal coronary veins closest to non‐scarred LMA segments in an image guided group (*n* = 89) versus control group (LV lead implantation in the posterolateral region) (*n* = 93).^15^ The image guided group had significantly fewer non‐responders using clinical composite scores (26% vs. 42%; *p* = .02) with no difference in echocardiographic volumetric parameters.^15^ Truong et al. used dual source cardiac CT to predict clinical outcomes in 54 patients scheduled for CRT in the DIRECT study.^6^ Intra‐procedural image guidance was not used, however, implanting physicians were given pre‐implant knowledge of coronary venous anatomy in half the patients. Lead location concordant to regions of maximal wall thickness was associated with reduced MACE (*p* < .01), however, CT dyssynchrony metrics and myocardial scar assessment did not predict 6‐month CRT response and prior knowledge of coronary venous anatomy by CT did not reduce implant or fluoroscopy time.^6^ More recently, Nguyên et al. successfully integrated CS roadmaps acquired from cardiac CTAs, with LGE imaging from Cardiac MRI and electrocardiographic imaging (ECGI) into 3D CRT roadmaps in 14 patients undergoing CRT implantation.^16^ The LV lead was positioned outside scar in LMA regions determined from ECGI in 11 out of 14 patients; in the remaining three patients LV scar could not be avoided and in two patients cannulation of the target vein was not possible due to limited coronary venous anatomy.^16^ These results suggest promise in targeting optimal LV lead placement, however, this is potentially a time and resource heavy preprocedural planning exercise requiring two cross‐sectional imaging modalities with ECGI integration. Nguyên et al.^16^ did not report preprocedural imaging and data processing or planning time which is likely to be reasonably long and may limit its clinical utility in real‐world clinical practice. Furthermore, validation of the optimal pacing site was not performed using hemodynamic assessment.^16^ Whilst LV scar imaging acquired from CMR is currently more reliable and reproducible than CT, if this were to change then CT and ECGI integration would certainly be a viable and potentially robust option for guiding LV lead implantation into LMA segments outside scar.

### 4.3 Intraprocedural cardiac CT versus cardiac MRI guidance for CRT and future directions

We have previously demonstrated feasibility of “real‐time” X‐MRI guidance.^3^ The advantage of cardiac CT over CMR for upgrade procedures relates to concerns around imaging CIEDs. MRI with non‐conditional devices is not without risk in pacing dependent patients (44% were pacing dependent in the present study). Moreover, significant image degradation with pre‐existing CIEDs limits CMR. There is also very little risk of claustrophobia with CT, scan times are markedly quicker and minimal breath holding is required, an important consideration in patients with heart failure who often struggle when supine for extended periods with repeated breath holds during CMR which may lead to significant image degradation. Cardiac CT also has the advantage of rapid imaging of coronary venous anatomy with submillimetre resolution which may determine whether suitable veins subtend target segments before implantation. LGE imaging with CMR is superior to late iodine enhancement with cardiac CT although there is a risk of nephrogenic systemic fibrosis with gadolinium contrast agents, particularly in renal impairment which is common in the heart failure population. Integration of multiple imaging modalities has been utilized,^16^ however this may add significant cost and time which may limit its application to clinical care. CMR‐guided LV lead implants may be best suited to patients undergoing de novo image guided CRT implantation with CT guidance being more appropriate for patients with pre‐existing devices. The TACTIC CRT trial is currently recruiting patients to compare intraprocedural CMR image overlay guidance to standard CRT implantation in patients with ischemic cardiomyopathy (NCT03992560). If late iodine enhancement protocols for cardiac CT improve and match CMR scar imaging reliability, then we could envisage that CT may become the imaging modality of choice for both de novo and upgrade procedures.

### 4.4 Study limitations

This is a small proof of principle study and larger multicentre, randomized controlled studies would be necessary to evaluate whether intraprocedural CT image overlay guidance is superior to standard care in patients undergoing CRT upgrades. We acknowledge that 17 out of 18 patients in this study were male which may have influenced the acute hemodynamic outcomes, however this is unlikely to have affected the primary feasibility outcomes. We did not perform statistical analysis on chronic outcomes in patients receiving LV leads delivered into the target versus nontarget segment due to small numbers of patients receiving an LV lead in the nontarget segment. LV dyssynchrony was computed using motion tracking of endocardial surfaces and used to identify LMA segments as opposed to strain calculation with myocardial tagging that could potentially neglect passive wall motion, however, similar tracking algorithms using both cardiac CT and CMR have shown good agreement with strain derived from myocardial tagging.^5^, ^17^ Furthermore, mechanical dyssynchrony was assessed during RV pacing and it is unknown whether analyzing mechanical dyssynchrony during RV pacing for defining the optimal pacing site is comparable to analyzing patients in intrinsic rhythm and further work in this area is warranted. The temporal resolution of cardiac CT in this study (up to 66 ms) remains inferior to echocardiography (20 ms) and CMR (35–50 ms obtained over multiple heart beats) and therefore cardiac CT may be less sensitive to subtle regional motion changes. Additionally, the predicted target vein derived from cardiac CT was either a lateral or posterolateral vein and a larger study would be required to assess the frequency of predicted alternative CS branches and evaluate the effect on clinical and echocardiographic outcomes. Furthermore, successful LV lead delivery was achieved to the CT‐derived target vein (89%) and target segment (83%). Whilst we believe these numbers are good for an image guidance approach, this remains a limitation of using a transvenous approach via the CS for LV lead delivery. This may also be seen as an advantage in preprocedural planning; if we are able to reliably predetermine whether there is absence of a suitable caliber vein subtending the target LV segment, then it could also be used to identify patients more suited to first‐line endocardial LV lead implantation. LV endocardial pacing may be useful in non‐responders to conventional CRT, however, the optimal site of stimulation varies greatly between patients.^18^ A CT guidance system may therefore help identify which patients are more suited to CT‐guided endocardial LV lead stimulation.^19^

CT‐guided CRT is resource and time intensive and undoubtedly more costly than using intraprocedural late electrical activation,^20^, ^21^ however the additional preprocedural anatomical planning and mechanical dyssynchrony data available with a CT‐guided approach offers the potential to avoid late electrically activating segments within scarred myocardium and target the latest mechanically and/or electrically activating segments outside of scar. Integration of these techniques may prove advantageous and further studies are required. In the present study, cardiac CT did not reliably identify late iodine enhancement irrespective of the imaging protocol used, however, regional hypoperfusion (hypoattenuating areas) was observed in regions of known LV scar from historical MRI data of the same patients. This raises the question whether all scar actually hyper‐enhances with iodinated contrast which may potentially also be seen as hypoattenuating areas in advanced scar formation. Dual energy scanning was used initially aiming for superior late iodine enhancement detection, however, scar identification proved unreliable. The protocol was modified to a single energy shuttle mode dynamic scan at 12.5 min balancing low image noise and low kVp required for late iodine detection with slight improvement. More research into accurate LV scar detection using cardiac CT is required with standardization of CT scar imaging protocols. Additionally, use of cardiac CT may not be feasible in all patients due to significant renal impairment or contrast allergy and given the small risks of ionizing radiation, this may not be an appropriate imaging modality for all patients.

## 5 CONCLUSION

Intraprocedural cardiac CT image overlay guidance for optimal LV lead placement in CRT upgrades is feasible with superior acute hemodynamics when pacing in CT target segments and favorable volumetric response rates, despite a high proportion of patients with ischemic cardiomyopathy. Multicentre, randomized controlled studies are needed to evaluate whether intraprocedural CT image overlay guidance to avoid scar and target late activating regions is superior to standard care.

## Supporting information

Supporting information.
