# Supplementary material for: Feasibility of intraprocedural integration of cardiac CT to guide left ventricular lead implantation for CRT upgrades
Source: J Cardiovasc Electrophysiol. 2021 Feb 10;32(3):802–12. doi: 10.1111/jce.14896 (PMC8647921; doi:10.1111/jce.14896)
Supplement: Supplementary file 1 — Supporting information. [file JCE-32-802-s001.docx]

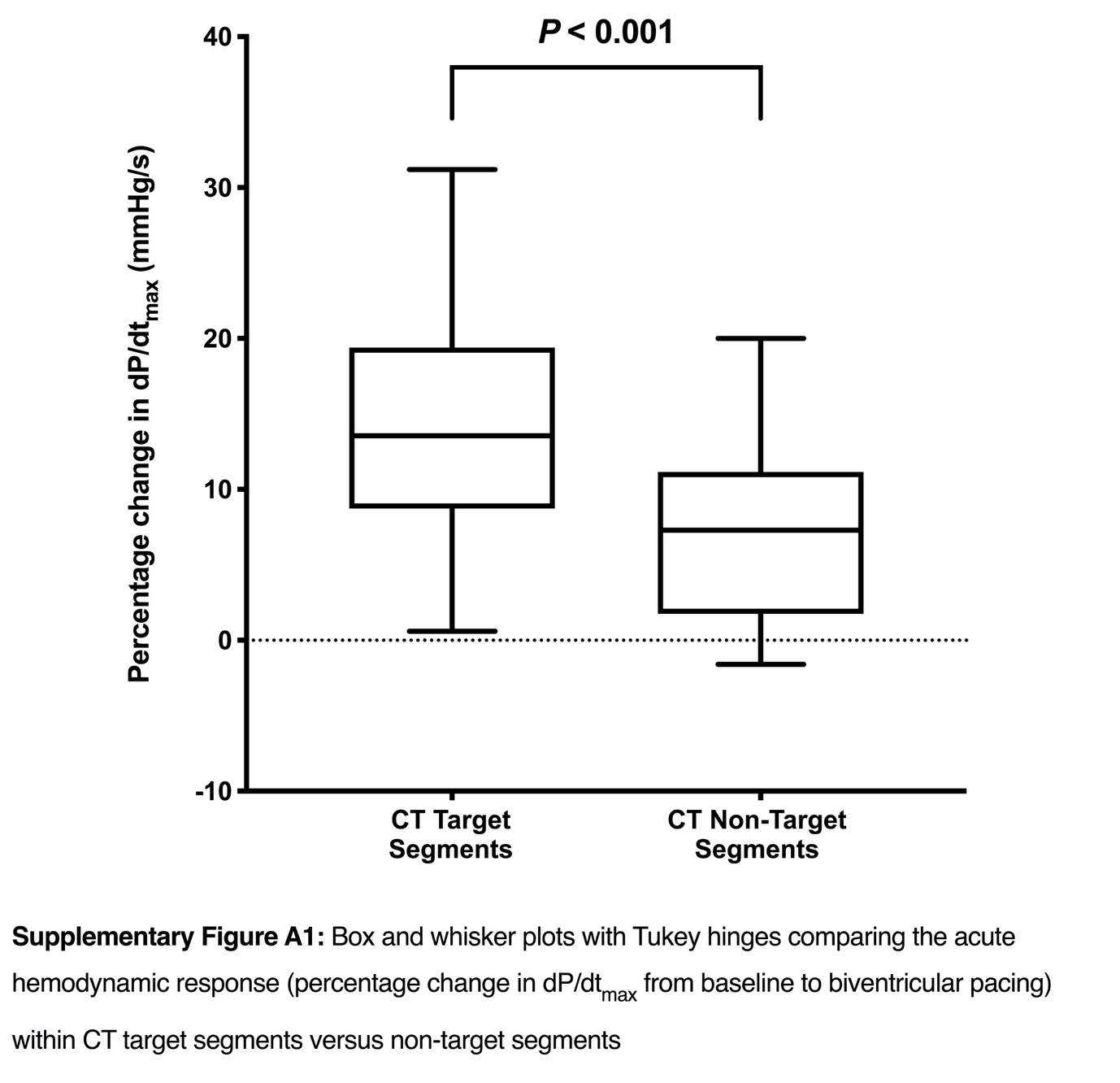


**Supplementary Table A1: CT defined scar characteristics and distribution in patients with ischemic cardiomyopathy**

| CT Scar characteristics | Value (n=10) |
| --- | --- |
| True late iodine enhancement | 2 (20%) |
| Hypoattenuation suggestive of hypoperfusion | 2 (20%) |
| Inferred scar by LV wall thinning, LV hypokinesis/akinesis | 6 (60%) |
| LAD artery territory**^a^** | 5 (50%) |
| LCX artery territory**^a^** | 5 (50%) |
| RCA artery territory**^a^** | 5 (50%) |
| Mean number of AHA CT defined scarred segments**^b^** | 3.9 ± 1.2 |

Values are presented as mean ± SD or as n (%).

LV=left ventricular, LAD=Left Anterior Descending Artery, LCx=Circumflex artery, RCA=Right coronary artery, AHA=American Heart Association, Computed Tomography

**^a^** 5/10 patients (50%) had more than 1 coronary artery territory involved

**^b^** Number of scarred segments includes all 10 patients with ischemic cardiomyopathy with CT evidence of left ventricular scar including late iodine enhancement, hypoattenuating areas suggestive of hypoperfusion and inferred scar indicated by left ventricular wall thinning and hypokinesis/akinesis.
